# The Exposure to Osteoarthritic Synovial Fluid Enhances the Immunomodulatory Profile of Adipose Mesenchymal Stem Cell Secretome

**DOI:** 10.1155/2020/4058760

**Published:** 2020-07-18

**Authors:** Adriana Cifù, Rossana Domenis, Massimo Pozzi-Mucelli, Paolo Di Benedetto, Araldo Causero, Massimo Moretti, Marta Stevanato, Cinzia Pistis, Pier Camillo Parodi, Martina Fabris, Francesco Curcio

**Affiliations:** ^1^Dipartimento di Area Medica, Università degli Studi di Udine, Udine, Italy; ^2^Istituto di Patologia Clinica, ASUFC, Udine, Italy; ^3^Dipartimento di Ortopedia e Traumatologia, ASUFC, Udine, Italy; ^4^DAME Università degli Studi di Udine, Clinica Ortopedica, ASUFC, Udine, Italy; ^5^VivaBioCell S.p.A., Udine, Italy; ^6^DAME Università degli Studi di Udine, Clinica di Chirurgia Plastica, ASUFC, Udine, Italy

## Abstract

**Objective:**

Several clinical studies have proposed the infusion of adipose mesenchymal stem cells (AMSCs) as an alternative therapy for joint diseases with inflammatory components, such as osteoarthritis. Indeed, AMSCs are able to stimulate tissue repair through a paracrine activity and the interaction with the inflammatory microenvironment seems to have a critical role.

**Design:**

To reproduce the inflammatory microenvironment, AMSCs were exposed to osteoarthritic synovial fluid (SF) for 48 h and the effect of their secretome on differentiation of monocytes (M0) into macrophages M1-like and mature dendritic cells (mDCs) was evaluated. Furthermore, the effect of the secretome of AMSCs exposed to SF was evaluated on the T cell population in terms of T cell proliferation and expansion of T regulatory cells (T reg).

**Results:**

Our data show that the exposure of AMSCs to SF activates cells and promotes the release of immunosuppressive factors, which induce macrophage polarization of M0 into the M2-like phenotype and inhibit differentiation of monocytes into mature dendritic cells (mDCs). Only the secretome of exposed AMSCs was able to inhibit T cell proliferation and promote T reg expansion.

**Conclusions:**

Our results suggest that the microenvironment plays a fundamental role for the development of anti-inflammatory and immunomodulatory properties of AMSCs.

## 1. Introduction

Mesenchymal stem cells (MSCs) are multipotent stem cells with self-renewal capability [1], which are widely distributed in a great number of adult and perinatal tissues, including bone marrow, adipose tissue, umbilical cord, placenta, amniotic fluid, liver, thymus, spleen, and gingiva [2]. Furthermore, MSCs possess strong genomic stability and can be isolated from their resident tissue and expanded in culture over several generations. Mesenchymal stem cells are able to differentiate into various lineages, both mesodermal and nonmesodermal cells [1, 3], a feature that contributes to their potential use in regenerative medicine [2]. A large number of clinical trials have been conducted or are ongoing to investigate MSCs as a potential therapy for a wide range of diseases [3], including acute myocardial infarction [4], spinal cord injury [5], and bone and joint diseases [6–8].

The main mechanisms associated with the therapeutic effects of MSCs include their ability to differentiate and replace damaged cells [9], and their paracrine [10, 11] and immunomodulatory activity on adjacent cells promoting tissue renewal [12]. However, several studies reported that MSCs disappear from the target tissue quickly after administration; therefore, the possibility that these cells exert their regenerative effects through differentiation to replace damaged cells appears to be a rare event *in vivo* [2]. Therefore, it is possible to hypothesize that soluble factors secreted by MSCs can help recover tissue homeostasis [10–12].

The immunomodulatory properties of MSCs were first described in 2002: Di Nicola et al. demonstrated that MSCs were able to inhibit the proliferation of T cells [13]. Subsequently, an increasing number of studies have demonstrated that MSCs have the ability to modulate both innate and adaptive immunity by suppressing dendritic cell maturation, inhibiting the proliferation of Natural Killer (NK) cells, promoting the generation of regulatory T cells, reducing the activation and proliferation of B cells, shifting macrophage differentiation from M1 to M2 macrophages, and suppressing T cell activity [14, 15].

The exact mechanisms by which MSCs are able to modulate the immune response are still not fully understood, but it is clear that cell-to-cell contact and the release of soluble factors, such as indoleamine-2,3-dioxygenase (IDO), nitric oxide (NO), prostaglandin-E2 (PGE-2), interleukine-10 (IL-10), and transforming growth factor-*β* (TGF-*β*) [1, 16] are involved.

It should be noted that the immunosuppressive capacity of MSCs is influenced by the inflammatory microenvironment [8, 17]. MSCs cultured in the presence of interferon-*γ* (IFN-*γ*), tumor necrosis factor-*α* (TNF-*α*), and IL-6 have been shown to increase their immunosuppressive capacity by releasing soluble factors [16]. Németh et al., using a mouse model of sepsis, demonstrated that bone marrow stromal cells (BMSCs) preactivated with lipopolysaccharides (LPS) or TNF-*α* are able to stimulate the production of IL-10 (the cytokine responsible for increasing animal survival rates in this model) by host macrophages [14]. On the other hand, an inflammatory environment is more tolerated by MSCs than priming with proinflammatory cytokines: equine bone marrow MSCs maintained their ability to proliferate and differentiate when exposed to inflammatory synovial fluid, while treatment with specific cytokines negatively affected their viability and ability to differentiate [18].

Moreover, Bustos et al. showed that the anti-inflammatory characteristics of MSCs improved after *in vitro* activation with serum from patients with acute respiratory distress syndrome (ARDS), demonstrating that activated MSCs increased the production of IL-10 and IL-1RN (interleukin-1 receptor antagonist) [19].

Osteoarthritis (OA) is the most common form of degenerative arthritis, causing pain and long-term disability [20]. Osteoarthritis is characterized by progressive destruction of articular cartilage, subchondral bone lesions, and synovial changes. In patients with OA, chronic and low-grade inflammation also contributes to disease progression through the release of many inflammatory molecules into synovial fluid [7, 8, 18, 21, 22]. The proinflammatory cytokines and chemokines present in osteoarthritic synovial fluid activate monocytes/macrophages (M*Φ*s) and dendritic cells (DCs) that commonly infiltrate the OA joint [23–25]. Furthermore, activation of the T cells present in OA-affected joints by M*Φ*s and DCs leads to a worsening of inflammation [23–25]. Conventional OA treatments focus on inflammation reduction and pain control [20, 26], but in recent years, the MSC injection has been proposed as an alternative approach [7].

Several clinical trials have shown that intra-articular injection of MSCs has improved clinical outcomes, reducing pain and improving joint function [6, 27, 28].

Although it is well known that the use of stem cells contributes to better clinical outcomes in OA patients, the molecular mechanisms responsible for the clinical findings have not been clarified. Recently, it has been reported that the exposure of MSCs to the articular microenvironment, represented by the osteoarthritic synovial fluid, could modulate some of the stem cells' properties such as proliferation, migration, cytokine receptor expression, cytokine secretion, and inhibition of lymphocyte proliferation [29–31].

To better understand the impact of the articular microenvironment on the immunomodulatory properties of MSCs after injection into the joint where the inflammatory process takes place, we have exposed adipose mesenchymal stem cells (AMSCs) to synovial fluid taken from osteoarthritic joints and studied the immunomodulatory effect of their secretome on immune cells involved in disease progression: macrophages (M1-like and M2-like), dendritic cells, and T cells.

## 2. Methods

### 2.1. Adipose Mesenchymal Stem Cell Isolation and Culture

Adipose mesenchymal stem cells (AMSCs) were isolated from adipose tissue obtained by lipoaspirates from subcutaneous abdominal fat and characterized as previously described [32]. One lipoaspirate from each donor, for a total of three female donors who underwent mammary reconstruction (mean age 43.7 ± 7.5), was collected (after informed consent and after approval by the Regional Bioethics Committee of the Friuli Venezia Giulia Region: consent no. CRO-2016-30).

Briefly, lipoaspirates were first enzymatically dissociated with 0.05% collagenase II (Worthington) for 20 minutes at 37°C, centrifuged at 500 × *g* for 5 minutes, and filtered through a 70 *μ*m nylon mesh (Merck Millipore). Cells were maintained under 5 vol% CO_2_ at 37°C in minimum essential medium-*α* (MEM-*α*) supplemented with 10% FBS (Gibco), penicillin/streptomycin solution (10 ml/l), alanine/glutamine solution (2 mM), human epidermal growth factor (10 ng/ml), insulin solution (10 *μ*g/ml), 2-fosfo-L-ascorbic acid, trisodium salt (100 *μ*M), and dexamethasone (0.01 *μ*M) (all from Sigma-Aldrich) [33]. AMSCs were characterized by flow cytometry using hematopoietic negative markers (CD34 and CD45) and mesenchymal stem cell positive markers (CD29, CD73, CD90, and CD105) as described previously (data not shown) [34].

Cells from between passages 2 and 4 were used for the experiment. AMSCs were isolated according to Good Manufacturing Practice (GMP) [35].

### 2.2. Adipose Mesenchymal Stem Cell Exposure to Osteoarthritic Synovial Fluid

Osteoarthritic synovial fluid was obtained by needle aspiration from *n* = 15 patients (8 males 65.4 ± 8.8 years of age and 7 females 75.5 ± 5.6 years of age). Patients scheduled for primary-intention knee replacement surgery due to end-stage knee osteoarthrosis were recruited at the Orthopaedic Units of the Hospital of Tolmezzo. All patients gave written informed consent.

To clean samples, SFs were treated with 2 mg/ml bovine testicular hyaluronidase type I-S (Sigma-Aldrich) for 30 minutes, then centrifuged at 14,000 × *g* for 20 minutes. Supernatants (SF) were pooled, aliquoted, and stored at -80°C until use [31].

The concentration of cytokines and chemokines present in osteoarthritic synovial fluid were quantified with a magnetic bead-based multiplex assay (Bio-Plex Pro™ Human Chemokine Panel, 40-Plex #171AK99MR2, Bio-Rad Laboratories).

AMSCs of each donor were seeded in triplicate at a density of 15,000 cells/cm^2^, and the day after, pooled SFs were added to the media in 20% or 50% ratio for 24 h and 48 h. After the incubation, AMSCs were collected and cell viability was determined using the trypan blue exclusion method. No differences were observed in cell viability among these different conditions (data not shown), then we chose to expose AMSCs to 50% of SFs for 48 h. The conditioned medium used for functional experiments with immune cells was also collected after 48 h, centrifuged for 10 minutes at 14,000 × *g*, and stored at -80°C until use.

For flow cytometry analysis, AMSCs were fixed and permeabilized with the intracellular Fix/Perm solution kit (#88-8824-00, eBiosciences), incubated with 5 ng/*μ*l of FITC-conjugated indoleamine-pyrrole 2,3-dioxygenase (IDO) antibody (clone: eyedio; eBiosciences) for 15 min, and then rinsed twice with PBS. Flow cytometry was performed with FACSCalibur (Becton Dickinson), and the data was analysed using the Flowing Software.

To evaluate the effect of SFs on AMSCs' production of cytokines and chemokines, after 48 h of SF-exposure, cells were rinsed with PBS and the culture medium was replaced for 24 h. Supernatants were collected and stored at -80°C until use. The concentrations of cytokines and chemokines secreted by AMSCs were quantified in the cellular supernatants collected after 24 hours with a magnetic bead-based multiplex assay (Bio-Plex Pro™ Human Chemokine Panel, 40-Plex #171AK99MR2, Bio-Rad Laboratories). The concentration of TGF-*β*1 was measured in the cellular supernatants with a magnetic bead-based single-plex assay (R&D Systems #LTGM100) according to the manufacturers' instructions.

### 2.3. Monocyte Differentiation into M1-like and M2-like Macrophages or Dendritic Cells

Human PBMCs (peripheral blood mononuclear cells) were isolated from EDTA-uncoagulated blood of three anonymous blood donors (with ages ranging from 18 to 65 years) by gradient centrifugation (Ficoll-Paque Plus, GE Healthcare), and monocytes were purified by negative selection using a commercial kit (EasySep™ Human Monocyte Enrichment Kit, Negative Selection #19059, Stemcell Technologies) according to the manufacturers' instructions. Purity was over 90% as assessed by staining with anti-CD14-FITC (5 ng/*μ*l, clone 61D3, eBioscience) and flow cytometric analysis (FACSCalibur). All blood donors gave written informed consent.

For macrophage differentiation, CD14+ monocytes (M0) were seeded in triplicate for nine days in multiwell plates at 5 × 10^5^/cm^2^ in RPMI medium supplemented with 10% heat-inactivated fetal bovine serum (FBS), 1% glutamine, 1% pyruvate, 1% nonessential amino acid, 1% penicillin/streptomycin, 1% HEPES (all from EuroClone), and 100 ng/ml macrophage colony-stimulating factor (M-CSF, Peprotech) for M2-like differentiation or 100 ng/ml granulocyte-macrophage colony-stimulating factor (GM-CSF, Peprotech) for M1-like differentiation. The complete media were changed every 3 days.

For dendritic cell (DC) differentiation, CD14+ monocytes were seeded in triplicate in multiwell plates at 5 × 10^5^/cm^2^ in RPMI medium supplemented for six days with 50 ng/ml granulocyte-macrophage colony-stimulating factor (GM-CSF, Peprotech) and 50 ng/ml IL-4 (Peprotech) obtaining immature DCs (iDCs). The complete media were changed every 3 days. To obtain mature DCs (mDCs), on the seventh day, 40 ng/ml of lipopolysaccharide (LPS from *Escherichia coli* O55:B5, Sigma-Aldrich) was added for 48 hours to the iDCs.

To evaluate the influence of AMSCs' secretome on the differentiation of monocytes into M1-like or mDC cells, each conditioned medium of SF-exposed AMSCs (CM+SF) was added separately to the differentiation media of monocytes obtained from each donor at a 50% ratio (technical replicates *n* = 3).

As a control for the effect of SF present in the conditioned medium, SF alone, at a similar ratio as CM+SF, was added to parallel cultures. The complete media were changed every 3 days.

Differentiated M1-like and M2-like macrophages were collected with TrypLE™ Express detachment solution (Gibco) and characterized by flow cytometry for the expression of macrophage markers: cells were incubated for 15 min with anti-CD80-PE (clone 2D10.4, eBiosciences), anti-CD163-APC (clone eBioGHI/61, eBiosciences), and anti-HLA-DR-FITC (clone L243, BD Pharmingen™) and then rinsed two times with PBS. To detect the expression of intracellular Arginase I, cells were fixed and permeabilized with the Fix/Perm solution kit (#88-8824-00, eBiosciences), incubated with anti-Arginase I-FITC (clone P05089, R&D Systems) for 15 min and then rinsed twice with PBS.

To evaluate the concentration of TNF-*α* secreted by M1-like cells, on the ninth day, after treatment with AMSC-derived CM and SF, cells were rinsed with PBS and the culture medium was replaced with RMPI medium. Supernatants were collected after 24 h and stored at -80°C until use. The concentration of TNF-*α* was measured with a magnetic bead-based simplex assay (TNF-alpha Human ProcartaPlex™ Simplex Kit #EXP01A-10223-901, Thermo Fisher Scientific).

As for macrophages, differentiated DCs were collected with TrypLE™ Express detachment solution (Gibco) and characterized by flow cytometry for the expression of DC markers using anti-CD14-FITC (clone 61D3, eBiosciences), anti-CD83-APC (clone HB15e, eBiosciences), anti-CD123 (clone 7G3, BD Pharmingen™), anti-CD80-PE (clone 2D10.4, eBiosciences), and anti-HLA-DR-FITC (clone L243 BD).

All antibodies for flow cytometry were used at final concentration of 5 ng/*μ*l.

Flow cytometry was performed with the FACSCalibur (Becton Dickinson), and the data were analysed using the Flowing Software.

To evaluate the concentration of IL-10 secreted by mDCs, on the ninth day, after treatment with AMSC-derived CM and SF, cells were rinsed with PBS and the culture medium was replaced with RMPI medium. Supernatants were collected after 24 h and stored at -80°C until use. The concentration of IL-10 was measured with a magnetic bead-based simplex assay (IL-10 Human ProcartaPlex™ Simplex Kit #EXP01A-10215-901-901, Thermo Fisher Scientific).

In all experiments with immune cells, the control was represented by cells cultured in differentiation medium only.

### 2.4. T Cell Proliferation Assay

PBMCs, isolated from three anonymous blood donors (with ages ranging from 18 to 65 years), were labelled with 5 *μ*M CFSE (carboxyfluorescein succinimidyl ester, Invitrogen) in PBS with 0.1% bovine serum albumin for 10 minutes at 37°C, followed by immediate quenching with cold culture medium.

To evaluate the effect of AMSCs' secretome on PBMCs, 2 × 10^5^ cells resuspended in RPMI medium were preincubated for 24 hours with 50% of AMSC-derived CM and SF, then seeded in triplicate into 96-well plates with prebound 0.5 *μ*g/ml anti-CD3 (clone OKT3, eBiosciences) and 0.5 *μ*g/ml anti-CD28 (clone CD28.6, eBiosciences). After 3 days, *in vitro*-stimulated PBMCs were stained with 5 ng/*μ*l of anti-CD3-APC (clone HIT3a, BioLegend) and cell proliferation was tested with flow cytometry (FACSCalibur, Becton Dickinson). Data were analysed using the Flowing Software.

The percentage of proliferating cells was calculated on the peak measured in unstimulated T cells. Proliferation was expressed as fold change of the proliferating cells over stimulated control cells.

### 2.5. T Reg Proliferation Assay

CD4+ T lymphocytes were purified from PBMCs isolated from three anonymous blood donors (with ages ranging from 18 to 65 years) by negative selection using the Human CD4+ T Cell Enrichment Kit (#19052, Stemcell Technologies) according to the manufacturers' instructions. Purification was over 90% as assessed by staining with 5 ng/*μ*l of anti-CD4-FITC (clone RPA-T4, eBioscience) and flow cytometric analysis (FACSCalibur).

Isolated CD4+ T lymphocytes (2 × 10^5^) were resuspended in RPMI medium (control) or preincubated for 24 hours with 50% of AMSC-derived CM and SF, then seeded in triplicate into 96-well plates with prebound 0.5 *μ*g/ml anti-CD3 (clone OKT3, eBiosciences), 0.5 *μ*g/ml anti-CD28 (clone CD28.6, eBiosciences), and recombinant IL-2 at a concentration of 250 U/ml (Peprotech). After 3 days, *in vitro*-stimulated CD4+ T cells were stained with 5 ng/*μ*l of anti-CD25-APC (clone BC96, eBiosciences) and 5 ng/*μ*l of anti-FoxP3-PE (clone PCH101, eBioscience) and T reg proliferation was tested with flow cytometry (FACSCalibur, Becton Dickinson). The data were analysed using the Flowing Software.

### 2.6. Statistical Analysis

Data are reported as mean of three experiments ± standard deviation (SD). Statistical analysis has been performed using GraphPad Software (version 7). Data were tested for normal distribution using the Kolmogorov-Smirnov test. For the data on AMSCs and AMSC+SF, paired *t*-test or nonparametric paired Wilcoxon's test, as appropriate, was used to compare continuous variables between two groups.

For all other experiments, repeated measurements were analysed by one-way ANOVA analysis of variance followed by the Bonferroni posttest. *P* values < 0.05 was considered significant.

## 3. Results

### 3.1. Effect of Osteoarthritic Synovial Fluid on Adipose Mesenchymal Stem Cells (AMSCs)

Osteoarthritic synovial fluids were characterized in terms of cytokines and chemokines by multilplex assay (Table 1).

The presence of cytokines and chemokines has been evaluated in the osteoarthritic synovial fluids by a magnetic bead-based 40-plex assay. Data are presented as the mean ± S.D. (*n* = 3). CCL: chemokine C-C motif ligand; CXCL: chemokine C-X-C motif ligand; CX3CL1: chemokine C-X3-C motif ligand 1; IL: interleukin; IFN-*γ*: interferon *γ*; GM-CSF: granulocyte-macrophage colony-stimulating factor; MIF: macrophage migration inhibitor factor; TNF-*α*: tumor necrosis factor *α*.

**Figure 1:**
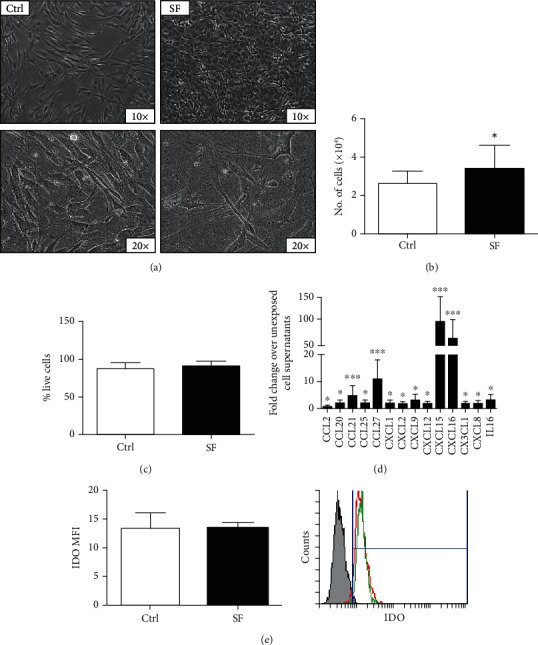
Exposure to osteoarthritic synovial fluid increases cell proliferation and promotes the release of cytokines and chemokines by AMSCs. (a) Representative phase contrast images (10x and 20x magnification) of AMSCs incubated without (CTRL) or with osteoarthritic synovial fluid (SF) for 48 hours. (b and c) Number of viable cells and viability were determined by the trypan blue exclusion assay. (d) The release of cytokines and chemokines of AMSCs after incubation with SF was measured in cell supernatants by a magnetic bead-based 40-plex assay. (e) IDO expression was determined by flow cytometry and reported as mean fluorescence intensity (MFI); histogram overlay shows isotype control staining (grey) versus specific antibody staining profile (green for control and red for AMSCs exposed to SF). Data are shown as mean (*n* = 3) ± S.D.^∗^Difference with untreated cells (*P* < 0.05). ^∗∗^Difference with untreated cells (*P* < 0.01). ^∗∗∗^Difference with untreated cells (*P* < 0.001).

To evaluate the effect of SF on AMSCs, cells were cultured in medium containing 50% SF. The addition of SF induced morphological changes in AMSCs, which became more elongated with an irregular shape (Figure 1(a)), and increased the number of viable cells (Figure 1(b)); however, the percentage of viable cells was not affected (Figure 1(c)). To study the effect of osteoarthritic SF on cytokine and chemokine production by AMSCs, cell supernatant was collected and analysed by a magnetic bead-based 40-multiplex assay. Concentrations of cytokine/chemokines secreted from untreated cells are reported in Table 2 (supplementary material). Exposure to osteoarthritic SF significantly upregulated (**P** < 0.05) the release of several cytokines/chemokines by AMSCs (Figure 1(d)). It should be noted that the production of CCL21, CCL27, CXCL15, and CXCL16 chemokines was more strongly influenced by the exposure of cells to SF (**P** < 0.001). Finally, the expression of the IDO immunosuppressive factor by AMSCs was not significantly affected by exposure to SF (Figure 1(e)).

### 3.2. Effect of Conditioned Medium of SF-Exposed AMSCs on Differentiation of Macrophages

**Figure 2:**
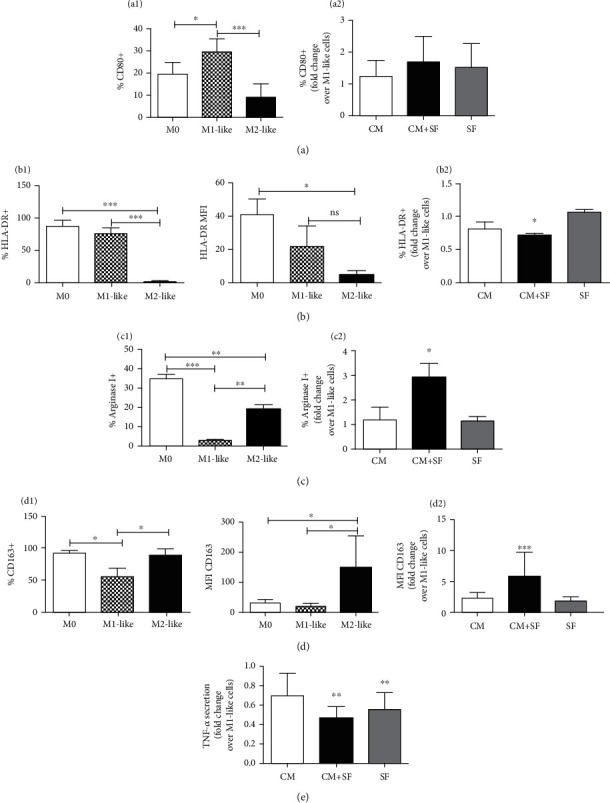
Conditioned medium of SF-exposed AMSCs reverts the M1 phenotype and promotes the polarization of macrophages into the M2 phenotype. Monocytes (M0) were differentiated into macrophages in the presence of GM-CSF (M1-like) or M-CSF (M2-like). The expression of CD80 ((a)1), HLA-DR ((b)1), Arginase I ((c)1), and CD163 ((d)1) was evaluated by flow cytometry. Conditioned medium of unstimulated (CM) or SF-exposed AMSCs (CM+SF) or synovial fluid only (SF) was added during M1-like differentiation. The expression levels of M1-like ((a)2 and (b)2) and M2-like markers ((c)2 and (d)2) are presented as fold increase compared to untreated M1-like cells. The secretion of TNF-*α* in M1-like macrophages treated with CM, CM+SF, and SF is expressed as fold increase with respect to the secretion of TNF-*α* in untreated M1-like cells. Data are shown as mean (*n* = 3) ± S.D.^∗^Difference with untreated cells (*P* < 0.05). ^∗∗^Difference with untreated cells (*P* < 0.01). ^∗∗∗^Difference with untreated cells (*P* < 0.001).

The CD14+ monocytes were induced for 9 days to differentiate into M1-like or M2-like macrophages with GM-CSF or M-CSF, respectively, and the expression of M1-like markers (CD80 and HLA-DR) and M2-like markers (intracellular Arginase I and CD163) was evaluated by flow cytometry. As expected, compared to monocytes (M0), the percentage of CD80-positive cells, as well as the mean fluorescence intensity (MFI) (data not shown), was increased after differentiation of macrophages towards the M1-like phenotype and was reduced in M2-like macrophages (Figure 2(a)1). The percentage of HLA-DR compared to M2-like cells was significantly higher both in M0 and in M1-like cells, and no difference was observed in MFI among M1-like and M2-like cells (Figure 2(b)1).

The expression, as well as the MFI (data not shown), of intracellular Arginase I was higher in M0 and in M2-like cells, while it was significantly reduced in M1-like cells (Figure 2(c)1). Finally, the percentage of CD163-positive cells was higher both in M0 and in M2-like cells and no difference was observed between these cells. However, MFI allows distinguishing the M2-like population from the M0 population (Figure 2(d)1); therefore, we chose to report the expression of CD163 as MFI.

To study the ability of AMSCs' secretome to induce an anti-inflammatory phenotype in macrophages, monocytes were differentiated in M1-like macrophages in the presence of AMSC-conditioned medium of unstimulated (CM) or SF-exposed AMSCs (CM+SF) or synovial fluid only (SF). Compared to M1-like cells, the expression of HLA-DR was significantly reduced (*P* < 0.05) only in M1-like cells treated with conditioned medium of SF-exposed AMSCs (Figure 2(b)2), while the expression of CD80 was not affected by treatment with AMSC-conditioned medium (Figure 2(a)2).

Furthermore, M1-like cells treated with CM+SF showed an increase of M2-like markers: Arginase I (Figure 2(c)2) and CD163 (Figure 2(d)2). A slight, but not significant effect was observed for conditioned medium of unstimulated AMSCs.

To confirm the ability of CM+SF to reverse the M1-like phenotype and promote the polarization of macrophages into M2-like cells, we evaluated the secretion of TNF-*α* in M1-like macrophages treated with conditioned medium of AMSCs: compared to M1-like cells, treatment with CM+SF and SF reduced the amount of this cytokine. No difference was observed among these two treatments.

### 3.3. Effect of Conditioned Medium of SF-Exposed AMSCs on Differentiation of Dendritic Cells

Monocytes were induced to differentiate into immature dendritic cells (iDCs) by stimulation with GM-CSF and IL-4, and to complete maturation (mDCs) with the addition of LPS.

To distinguish M0 from iDCs and mDCs, several markers were evaluated by flow cytometry.

**Figure 3:**
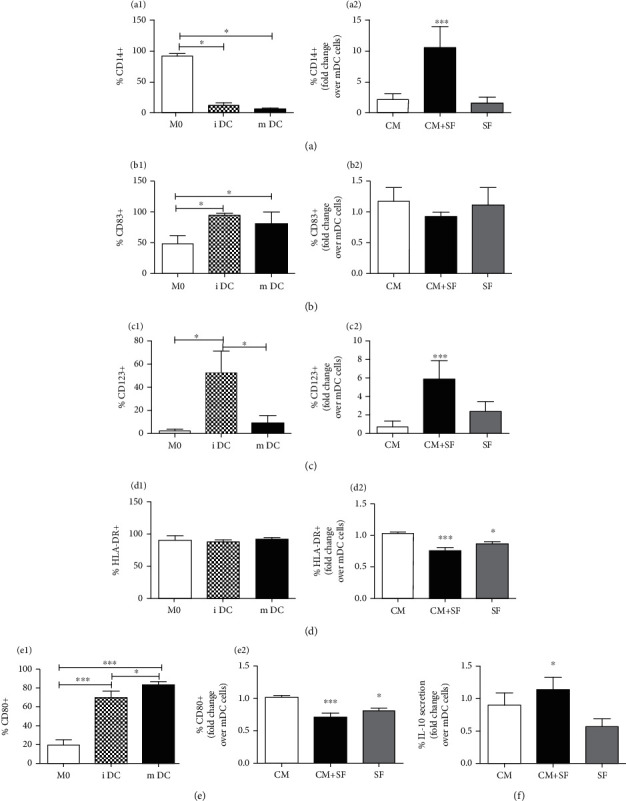
The conditioned medium of SF-exposed AMSCs inhibits differentiation of monocytes into DCs. Monocytes (M0) were differentiated into dendritic cells (DCs) in the presence of GM-CSF/IL-4 and LPS. To characterize dendritic cells, the expression of CD14 ((a)1), CD83 ((b)1), CD123 ((c)1), HLA-DR ((d)1), and CD80 ((e)1) was evaluated by flow cytometry. Conditioned medium of unstimulated (CM) or SF-exposed AMSCs (CM+SF) or synovial fluid only (SF) was added during DC differentiation. The expression levels of dendritic cells' markers are presented as fold increase compared to untreated mDCs ((a)2, (b)2, (c)2, (d)2, and (e)2). The secretion of IL-10 in mDCs treated with CM, CM+SF, and SF is expressed as fold increase with respect to the secretion of IL-10 in untreated mDCs. Data are shown as mean (*n* = 3) ± S.D.^∗^Difference with untreated cells (*P* < 0.05). ^∗∗^Difference with untreated cells (*P* < 0.01). ^∗∗∗^Difference with untreated cells (*P* < 0.001).

Both iDCs and mDCs expressed a low level of CD14 (Figure 3(a)1) and a high level of CD83 (Figure 3(b)1). The expression of CD123 was significantly higher only in iDCs (Figure 3(c)1). No difference was observed in HLA-DR expression among M0, iDCs, and mDCs. Finally, CD80 was differently expressed in all three cell populations (Figure 3(e)1).

Then, we examined the effect of the conditioned medium of unstimulated (CM) or SF-exposed AMSCs (CM+SF) or synovial fluid alone (SF) on DC differentiation. Our data showed that the conditioned medium of SF-exposed AMSCs inhibited differentiation of monocytes into mDCs: the treatment with CM+SF induced an increment of CD14 and CD123 expression (*P* < 0.05). Both CM+SF and SF treatments reduced the expression of HLA-DR and CD80. Neither treatment influenced the expression of CD83.

We evaluated the secretion of IL-10, a typically cytokine secreted by tolerogenic dendritic cells, in mDCs treated with conditioned medium. Compared to untreated mDCs, CM+SF induced an increase of IL-10 secretion (*P* < 0.05). No effect was observed in mDCs treated with CM or SF (Figure 3(f)).

### 3.4. Effect of Conditioned Medium of SF-Exposed AMSCs on T Cell Proliferation

**Figure 4:**
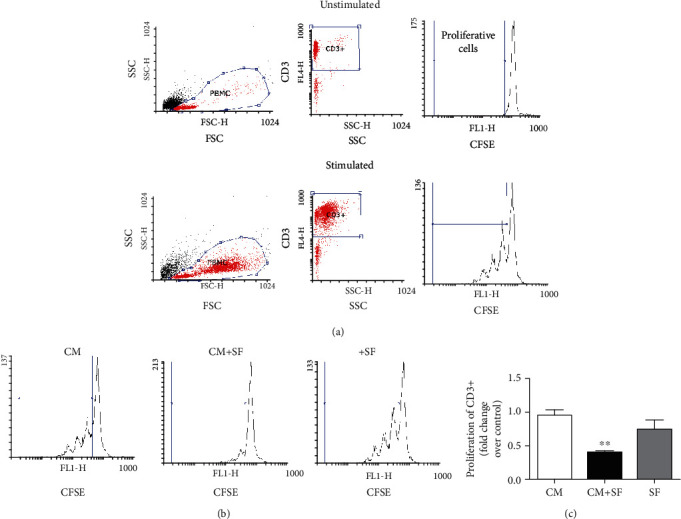
The conditioned medium of SF-exposed AMSCs inhibits the proliferation of CD3+ T cells. Gating strategies of T cells in proliferation assay—physical parameters, i.e., forward scatter (FSC) and side scatter (SSC), were used to select PBMCs. T cells were recognized by evaluating in PBMCs the expression of CD3. Proliferation was expressed as fold change of the proliferative cells with respect to stimulated control cells (a). CFSE-labelled PBMCs isolated from healthy donors were incubated in the presence of conditioned medium of unstimulated (CM) or SF-exposed AMSCs (CM+SF) or synovial fluid only (SF). (b) Representative CFSE cytometry histograms. (c) The histograms show the proliferation of CD3+ cells, expressed as fold increase compared to untreated cells. Data are shown as mean (*n* = 3) ± S.D.^∗∗^Difference with untreated cells (*P* < 0.01).

Unstimulated cells show a single, bright CFSE fluorescence peak, indicating no proliferation while stimulated cells show multiple CFSE fluorescence peaks, indicating multiple generations of proliferating cells (Figure 4(a)).

Conditioned medium collected after culturing AMSCs in 50% SF was used to analyse the effect of AMSCs' secretome on the proliferation of T cells (Figure 4). Conditioned medium of untreated AMSCs did not affected the proliferation of CD3+ T cells, while the conditioned medium of SF-exposed AMSCs, as shown above, caused significant inhibition. Treatment of PBMCs with synovial fluid (SF) alone had no effect on cell proliferation (Figures 4(b) and 4(c)).

### 3.5. Effect of Conditioned Medium of SF-Exposed AMSCs on T Reg Proliferation

**Figure 5:**
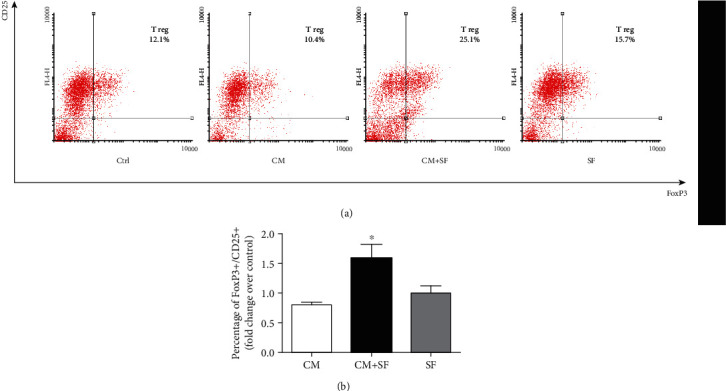
The conditioned medium of SF-exposed AMSCs increased the expansion of T reg. CD4+ T cells, isolated from PBMCs by negative selection, were stimulated with anti-CD3, anti-CD28, and IL-2 in the presence of conditioned medium of untreated cells (CM), conditioned medium of AMSCs exposed to synovial fluid (CM+SF), and synovial fluid only (SF). The percentage of regulatory T cells (CD4+/CD25+/FoxP3+) was determined by fluorescence-activated flow cytometry (FACS) on day 4. Representative plots for CD25 and FoxP3 staining are shown (a), and histograms represent the percentage of regulatory T cells expressed as fold increase over untreated cells (CTRL). Data are shown as mean (*n* = 3) ± S.D.^∗^Difference with the untreated cells, *P* < 0.05.

Conditioned medium collected after culturing AMSCs in 50% SF was used to analyse the effect of AMSCs' secretome on the expansion of T reg (Figure 5). Compared to untreated CD4+ cells (control), treatment of CD4+ with SF and CM+SF promoted the expansion of T reg cells, but only in the presence of CM+SF did the expansion of T reg increase significantly (*P* < 0.05). Conditioned medium of untreated AMSCs did not affected the proliferation of T reg.

## 4. Discussion

The ability of AMSCs to secrete a variety of trophic factors with different functions has motivated interest in evaluating their local or systemic injection to stimulate tissue repair in various diseases, including joint inflammatory diseases [36]. Clinical trials have shown that local injection of AMSCs into an osteoarthritic joint has improved function and is likely to play several roles, such as inhibiting osteophyte formation and reducing cartilage degeneration [7, 37].

The anti-inflammatory properties of AMSCs have been linked to their cell-cell-mediated immunosuppressive potential in collaboration with the secretion of soluble immune factors [38]. These modulators included a multitude of soluble immunomodulating factors, such as cytokines and growth factors, and extracellular vesicles [32, 39].

Recent studies have revealed that the immunomodulatory properties of AMSCs are not constitutive, but rather activated by signals derived from a proinflammatory microenvironment. In particular, AMSCs require “licensing” by proinflammatory cytokines to acquire an immunosuppressive phenotype [40]. Indeed, AMSCs' secretome is influenced by mutual interaction with immune cells [41] and is affected by specific disease-related tissue microenvironments. However, so far, AMSCs' secretome has been analysed after cytokine treatment, in order to make the stimulus more reproducible. IFN-*γ* in combination with one of the proinflammatory cytokines, TNF-*α*, IL-1*α*, or IL-1*β*, can stimulate MSCs to release high concentrations of immunosuppressive factors, as well as a burst of chemokine and adhesion molecule expression [42]. In this context, recent studies are aimed at developing strategies to guide the MSC secretome towards a more anti-inflammatory and regenerative phenotype [43, 44].

The osteoarthritic synovial fluid best represents the microenvironment of an inflamed joint. Indeed, our data demonstrated and confirmed [22, 45] the presence of proinflammatory molecules in osteoarthritic synovial fluid. As already reported [29–31], osteoarthritic synovial fluid influences the expression of molecules involved in immunomodulation. Furthermore, Sayegh et al. recently demonstrated that coculture of AMSCs exposed to SF of patients affected by rheumatoid arthritis (RA), with activated monocyte or CD4+ cells, can inhibit the expression of CD40 and CD80 (monocyte's proinflammatory markers) and promote T reg expansion [46].

In our study, we analysed the effect of osteoarthritic synovial fluid on AMSCs; in particular, we have verified the hypothesis that osteoarthritic synovial fluid alters the therapeutic efficacy of AMSCs, influencing their immunomodulatory properties.

Osteoarthritic synovial fluid is well tolerated by equine bone-marrow-derived MSCs, which have maintained their viability, proliferation, and differentiation abilities [18, 47, 48], and have increased the expansion of human MSCs in tissue culture of the synovium from osteoarthritic patients as measured by cell migration [49]. In addition, we confirmed that exposure to osteoarthritic SF significantly upregulated the release of several chemokines (CCL21, CCL27, CXCL15, and CXCL16) involved in the homing of various immune cells such as T cells and neutrophils [50–53].

AMSCs constitutively secrete a multitude of different members of the chemokine family, leading to an accumulation of immune cells near MSCs, thus creating a microenvironment in which the effects of locally acting factors produced by MSCs are amplified [42].

Moreover, we reported that exposure to osteoarthritic SF modifies the secretome of AMSCs, making it capable of reversing the M1-like phenotype by promoting macrophage polarization into M2-like cells, inhibiting differentiation of monocytes into DCs, and reducing proliferation of T cells. Moreover, the increase of T reg cells that we showed may explain the inhibitory effect on CD3+ cells.

In agreement with our results, it has been reported that the coculture of macrophages with MSCs induces differentiation in M2 macrophages and prevents differentiation of monocytes treated with GM-CSF/IL-4- in DCs by metabolic reprogramming through lactate secretion [54].

It has also been reported that the maturation of DCs induced by LPS treatment was inhibited by MSCs in coculture but not by cell supernatants, even if MSCs were preactivated with inflammatory cytokines. The authors suggested that IFN-*γ* treatment is not sufficient to induce the release of immunomodulatory molecules from MSCs, which probably requires a more complex stimulation, comparable to that present in the inflammatory microenvironment [55, 56].

In our experiments, only the secretome obtained from MSCs exposed to osteoarthritic SF induces a significant effect on the immune cells used for the experiments. Our data suggest that the inflammatory molecules present in the osteoarthritic synovial fluid, such as TNF-*α*, IL-1*α*, IL-1*β*, IL-6, MMP-3, and MMP-9 (metalloproteinases 3 and 9) [21, 57] are all essential to induce stem cells to secrete immunomodulatory factors. Indeed, as demonstrated, T cell proliferation has been inhibited by MSCs only in the presence or proinflammatory cytokines [58]. Moreover, MSCs were activated only by strongly stimulated T cells [59].

In conclusion, our study suggests that exposure to osteoarthritic synovial fluid enhances the immunomodulatory properties of the AMSCs' secretome and promotes the anti-inflammatory profile of immune cells, further supporting the hypothesis that communication with the inflammatory microenvironment plays an essential role in determining the ability of AMSCs to suppress the immune response.

## Figures and Tables

**Table 1:** Cytokines and chemokines in SF.

Analyte	pg/ml	Analyte	pg/ml
CCL1	41.6 ± 8.8	CXCL5	145.5 ± 34.4
CCL2	338.3 ± 276.7	CXCL6	6.5 ± 0.4
CCL3	6.8 ± 2.6	CXCL9	171.2 ± 50.4
CCL7	44.4 ± 2.7	CXCL10	246.6 ± 51.3
CCL8	19.1 ± 6.7	CXCL11	6.5 ± 1.5
CCL11	20.7 ± 4.9	CXCL12	833.8 ± 58.7
CCL13	6.8 ± 1.2	CXCL13	11.3 ± 2.3
CCL15	4762.5 ± 501.3	CXCL16	925 ± 36.5
CCL17	12 ± 3.1	CX3CL1	42.1 ± 7.9
CCL19	92.8 ± 19.5	IL-1*β*	1.4 ± 0.5
CCL20	5.6 ± 0.7	IL-2	6.4 ± 1.5
CCL21	3333.1 ± 22.9	IL-4	15.1 ± 3.3
CCL22	183.3 ± 43	IL-6	68.2 ± 31.8
CCL23	390.2 ± 12.7	CXCL8	21.6 ± 10
CCL24	191.2 ± 61.6	IL-10	13.5 ± 1.6
CCL25	353 ± 54.3	IL-16	506.4 ± 34.1
CCL26	28.9 ± 8.3	IFN-*γ*	27.9 ± 6
CCL27	499.6 ± 211.2	GM-CSF	38.3 ± 4.9
CXCL1	87 ± 13	MIF	7846 ± 106.5
CXCL2	21.1 ± 5.8	TNF-*α*	22.6 ± 8.2

## Data Availability

The experimental data used to support the findings of this study are included within the article.

## Supplementary Materials

Supplementary Materials Table 2: cytokine and chemokine production by untreated AMSCs.
