# Supplementary material for: The Exposure to Osteoarthritic Synovial Fluid Enhances the Immunomodulatory Profile of Adipose Mesenchymal Stem Cell Secretome
Source: Stem Cells Int. 2020 Jul 18;2020:4058760. doi: 10.1155/2020/4058760 (PMC7383307; doi:10.1155/2020/4058760)
Supplement: Supplementary Materials — Table 2: cytokine and chemokine production by untreated AMSCs. [file 4058760.f1.docx]

**Supplementary material**


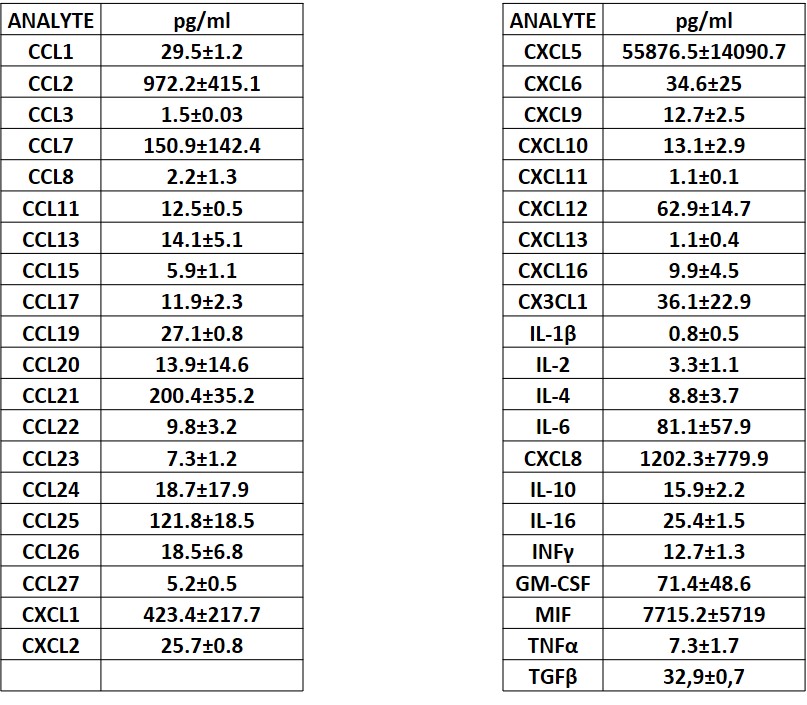


**Table 2. Cytokines and chemokines production by untreated AMSCs**

The production of cytokines and chemokines has been evaluated in the cell supernatants of AMSCs by magnetic beads-based 40 plex assay. The production of TGFβ has been evaluated in the cell supernatants of AMSCs by magnetic beads-based singleplex assay. Data are presented as the mean ± S.D. (n=3).

CCL (chemokine C-C motif ligand); CXCL (chemokine C-X-C motif ligand); CX3CL1 (chemokine C-X3-C motif ligand 1); IL (interleukin) ; INFγ (interferon γ); GM-CSF (granulocyte-macrophage colony-stimulation factor); MIF (macrophage migration inhibitor factor); TNFα (tumor necrosis factor α), TGFβ (transforming growth factor β).
